# Enhanced Tuberculosis Diagnosis With Computer-aided Chest X-ray and Urine Lipoarabinomannan in Adults With HIV Admitted to Hospital (CASTLE Study): A Cluster Randomized Trial

**DOI:** 10.1093/cid/ciae273

**Published:** 2024-05-15

**Authors:** Rachael M Burke, Saulos K Nyirenda, Timeo Mtenga, Hussein H Twabi, Elizabeth Joekes, Naomi F Walker, Rose Nyirenda, Ankur Gupta-Wright, Marriott Nliwasa, Katherine Fielding, Peter MacPherson, Elizabeth L Corbett

**Affiliations:** Faculty of Infectious and Tropical Disease, London School of Hygiene and Tropical Medicine, London, United Kingdom; Malawi Liverpool Wellcome Trust Clinical Research Programme, Blantyre, Malawi; Zomba Central Hospital, Ministry of Health, Zomba, Malawi; Faculty of Infectious and Tropical Disease, London School of Hygiene and Tropical Medicine, London, United Kingdom; Malawi Liverpool Wellcome Trust Clinical Research Programme, Blantyre, Malawi; Helse Nord Tuberculosis Initiative, Kamuzu University of Health Sciences, Blantyre, Malawi; Department of Clinical Sciences, Liverpool School of Tropical Medicine, Liverpool, United Kingdom; Department of Clinical Sciences, Liverpool School of Tropical Medicine, Liverpool, United Kingdom; Department of HIV/AIDS, Ministry of Health, Lilongwe, Malawi; Faculty of Infectious and Tropical Disease, London School of Hygiene and Tropical Medicine, London, United Kingdom; Institute for Global Health, University College London, London, United Kingdom; Malawi Liverpool Wellcome Trust Clinical Research Programme, Blantyre, Malawi; Helse Nord Tuberculosis Initiative, Kamuzu University of Health Sciences, Blantyre, Malawi; Faculty of Epidemiology and Population Health, London School of Hygiene and Tropical Medicine, London, United Kingdom; Faculty of Infectious and Tropical Disease, London School of Hygiene and Tropical Medicine, London, United Kingdom; Malawi Liverpool Wellcome Trust Clinical Research Programme, Blantyre, Malawi; School of Health and Wellbeing, University of Glasgow, Glasgow, United Kingdom; Faculty of Infectious and Tropical Disease, London School of Hygiene and Tropical Medicine, London, United Kingdom; Malawi Liverpool Wellcome Trust Clinical Research Programme, Blantyre, Malawi

**Keywords:** HIV, TB, hospitalization, diagnostics, advanced HIV disease

## Abstract

**Background:**

People with human immunodeficiency virus (PHIV) admitted to the hospital have high mortality, with tuberculosis (TB) being the major cause of death. Systematic use of new TB diagnostics could improve TB diagnosis and might improve outcomes.

**Methods:**

We conducted a cluster randomized trial among adult PHIV admitted to Zomba Central Hospital, Malawi. Admission days were randomly assigned to: enhanced TB diagnostics using urine lipoarabinomannan (LAM) antigen tests (SILVAMP-LAM, Fujifilm, Japan and Determine-LAM, Alere/Abbot, USA), digital chest X-ray with computer-aided diagnosis (dCXR-CAD, CAD4TBv6, Delft, Netherlands), plus usual care (“enhanced TB diagnostics”); or usual care alone (“usual care”). The primary outcome was TB treatment initiation during admission. Secondary outcomes were 56-day mortality, TB diagnosis within 24 hours, and undiagnosed TB at discharge, ascertained by culture of one admission sputum sample.

**Findings:**

Between 2 September 2020 and 15 February 2022, we recruited 419 people. Four were excluded postrecruitment, leaving 415 adults recruited during 207 randomly assigned admission days in modified intention-to-treat analysis. At admission, 90.8% (377/415) were taking antiretroviral therapy with a median CD4 cell count of 240 cells/mm^3^. In the enhanced diagnostic arm, median CAD4TBv6 score was 60 (interquartile range: 51–71), 4.4% (9/207) had SILVAMP-LAM–positive and 14.4% (29/201) had Determine-LAM–positive urine with 3 samples positive by both urine tests. TB treatment was initiated in 46/207 (22.2%) in the enhanced TB diagnostics arm and 24/208 (11.5%) in the usual care arm (risk ratio, 1.92; 95% confidence interval [CI]: 1.20–3.08). There was no difference in mortality by 56 days (enhanced TB diagnosis: 54/207, 26.1%; usual care: 52/208, 25.0%; hazard ratio. 1.05; 95% CI: .72–1.53); TB treatment initiation within 24 hours (enhanced TB diagnosis: 8/207, 3.9%; usual care: 5/208, 2.4%; risk ratio, 1.61; 95% CI: .53–4.71); or undiagnosed microbiological-confirmed TB at discharge (enhanced TB diagnosis, 0/207 [0.0%], usual care arm 2/208 [1.0%]; *P* = .50.

**Interpretation:**

Urine SILVAMP-LAM/Determine-LAM plus dCXR-CAD diagnostics identified more hospitalized PHIV with TB than usual care. The increase in TB treatment appeared mainly because of greater use of Determine-LAM, rather than SILVAMP-LAM or dCXR-CAD. Poor concordance between Determine-LAM and SILVAMP-LAM urine tests requires further investigation. Inpatient mortality for adults with human immunodeficiency virus remains unacceptability high.

## Teaser

A diagnostic intervention of screening with urine SILVAMP-LAM/Determine-LAM plus dCXR-CAD identified more hospitalized PHIV with TB than usual care. There was poor concordance between Determine-LAM and SILVAMP-LAM tests. Inpatient mortality for adults with HIV remains unacceptability high.

## Graphical Abstract

**Graphical Abstract:**
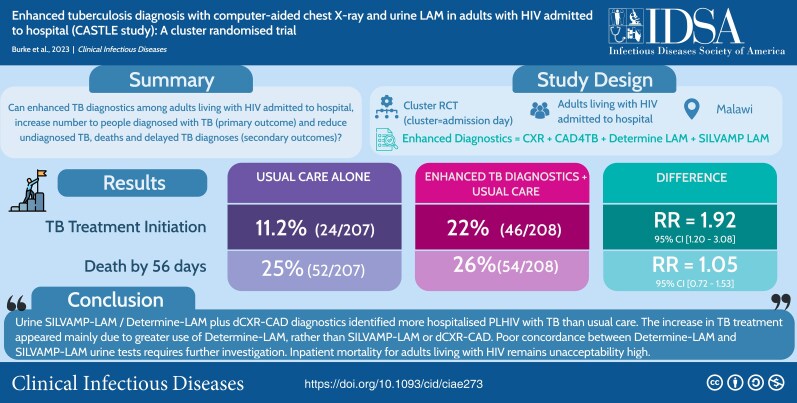
This graphical abstract is also available at Tidbit: https://tidbitapp.io/tidbits/enhanced-tuberculosis-diagnosis-with-computer-aided-chest-x-ray-and-urine-lam-in-adults-with-hiv-admitted-to-hospital-castle-study-a-cluster-randomised-trial-19b5123b-3106-4648-b3a2-68389a4cced1

People with human immunodeficiency virus (PHIV) who are admitted to the hospital are at very high risk of death during or shortly after hospital admission [1–3]. Tuberculosis (TB) is the most common reason for admission to hospital and inpatient death for PHIV [1, 4–7]. Difficulties in TB diagnosis contribute substantially to inpatient mortality, with many people dying from HIV-associated tuberculosis in autopsy studies not diagnosed before death, despite contact with health services [5, 6, 8–10]. Urine lipoarabinomannan (LAM) testing using lateral flow LAM (Determine-LAM, manufactured by Alere/Abbott, USA) has been shown in 2 trials to reduce 8-week all-cause mortality among high-risk groups admitted to the hospital [11–13]. However, Determine-LAM has suboptimal sensitivity [14, 15]. A newer LAM test (SILVAMP-LAM, manufactured by Fujifilm, Japan) was reported to have higher sensitivity than Determine-LAM [16].

Computer-aided diagnosis (CAD) on digital chest X-ray (dCXR-CAD) is as accurate as radiologists in diagnosing pulmonary TB [17, 18] and may shorten time to TB treatment initiation [19], although radiological screening studies and CAD product development have mainly focused on outpatient or community settings to date [20, 21]. Using dCXR-CAD to screen for likely pulmonary TB together with Determine-LAM and SILVAMP-LAM to diagnose disseminated TB may help to identify more people with TB among hospitalized PHIV, with potential to improve clinical outcomes and reduce mortality.

We therefore conducted a randomized trial to determine the effectiveness of enhanced TB diagnostics (using dCXR-CAD plus SILVAMP-LAM plus Determine-LAM) plus usual care on TB treatment initiation compared to usual clinician-directed testing among adults PHIV admitted to the hospital.

## METHODS

### Study Design and Participants

We conducted a cluster randomized trial in Zomba Central Hospital, Malawi. Each cluster was an admission day, covering a 24-hour period between 3:00 Pm and 2:59 Pm. Clusters were randomized in a 4:4:1 ratio to usual care alone, enhanced TB diagnostics plus usual care, or to a diagnostic cohort. Participants in clusters assigned to the diagnostic cohort are not reported here.

Inclusion criteria were that participants were adults (aged 18 years and older), diagnosed with HIV, admitted to medical wards at the Zomba Central Hospital less than 18 hours before recruitment to the trial, were willing and able to give consent, and who were not already taking treatment for TB disease or having completed TB treatment in previous 6 months. Potential participants were eligible for recruitment regardless of symptoms, reasons for admission, or antiretroviral therapy (ART) use. We used a cluster randomized design so that when usual care clinical staff reviewed people newly admitted to hospital, all eligible participants on a morning postadmission ward round would receive the same intervention, and because it was an efficient use of limited radiographer, transport, and laboratory staff resources.

### Randomization and Masking

The randomization sequence was computer generated, using block randomization with variable block size. An independent researcher generated the randomization codes and put them into sealed opaque envelopes. Once the cluster allocation was revealed by opening the envelope each morning, participants, study staff, and clinicians were not blinded to allocation. No interim analyses were done.

### Procedures

All participants able to produce sputum had 1 sample taken at recruitment for *Mycobacterium tuberculosis* culture. Sputum culture results were communicated to participants and routine clinical staff when available, but because culture results take up to 6 weeks, these did not affect in-hospital TB treatment decisions.

Participants admitted on a day assigned to the usual care arm could receive any tests usually available at Zomba Central Hospital. This included: nucleic acid amplification testing for TB (Xpert MTB/rifampicin [RIF], Cephid, USA) on sputum and other samples; X-ray with interpretation by radiographers, medical officers, or nonradiologist physicians; and urine testing with Determine-LAM. None of these tests was protocol-mandated; completion was dependent on request by routine clinical staff and tests were carried out by usual care staff—for Xpert, this was by hospital laboratory staff, for Determine-LAM, this was by medical assistants in wards. Malawi guidelines at the time recommended Determine-LAM for all HIV-positive inpatients, but individual usual care clinicians applied their own clinical judgment to request a test or not. All TB tests are free for all patients in the Malawi public health system.

Participants admitted on a day assigned to the enhanced TB diagnostics arm additionally received urine TB testing using SILVAMP TB-LAM and Determine-LAM, and a dCXR-CAD using CAD4TB v6 software (Delft imagining, Netherlands). The CAD4TB software gives a probabilistic score depending on how likely pulmonary TB is: scores are between 0 (very low chance of TB) and 100 (very high chance of TB). Users are responsible for setting their own threshold to determine actions; our threshold of 60 was determined before the trial start through review of X-rays from hospitalized patients (nontrial participants) and consensus discussion between trial investigators and an experienced radiologist. When the CAD4TB score was ≥60, and the participant could expectorate sputum [22], the study team would obtain a sputum sample for Xpert (sputum Xpert could also be requested by usual care teams in usual care). Study staff were trained in urine LAM testing and interpretation of results, and each trial-provided urine LAM test strip was read by 2 study staff members (nonblinded) to ensure consistency in test interpretation. After the completion of this trial, data from 2 multicountry studies were published showing substantial lot-to-lot variation in the accuracy of SILVAMP-LAM [22, 23]. In this trial, all SILVAMP-LAM tests were from batch numbers 19002 or 20004.

Test results were provided on a sticker in participants medical records, with chest X-ray available to view on screen. Decisions about TB treatment initiation were made by routine clinical staff.

### Outcomes

The primary outcome was TB treatment initiation before death or discharge from hospital (whichever happened first). The secondary outcomes were mortality up to 56 days from enrollment, TB treatment initiation within 24 hours of enrollment, and undiagnosed TB, defined by discharge or death without initiation of TB treatment in a participant whose admission sputum culture grew *M. tuberculosis*.

### Statistical Analysis

The sample size was 102 clusters (admission days) per trial arm. We anticipated a median cluster size of 3 participants per day, an intra-cluster correlation coefficient (ρ) of 0.005, and that in the control arm 10% of participants would initiate TB treatment [11]. A total of 102 clusters per trial arm gave 80% power to detect a difference between arms at least as large as a relative increase of 1.8, equivalent to an absolute increase of 8 percentage points.

Analysis was at the individual level, rather than cluster level. For analysis of the primary outcome (TB treatment initiation), we estimated a risk ratio and 95% confidence interval comparing trial arms using a log-binomial regression model, with robust standard errors to account for clustering. For the secondary outcome of TB treatment initiation within 24 hours we used log-binomial regression, and for the survival outcome we used a Cox regression model to estimate a hazard ratio—both with robust standard errors to account for clustering. For the undiagnosed TB at discharge outcome, we were unable to estimate a risk ratio because of having 0 events in 1 arm; we therefore tested for a difference between arms by calculating a 2-sided Fisher exact *P* value.

We had 2 prespecified subgroups for the primary outcome: people with TB in the differential diagnosis at admission and people with a CD4 count ≤100 cells/mm^3^. For all outcomes, we conducted a modified intention-to-treat analysis, with people found to be ineligible following recruitment removed from the outcome analysis. We did analyses in Stata (v15).

### Ethics, Funding, Trial Registration and Data Availability

We obtained ethical approval from London School of Hygiene & Tropical Medicine and the College of Medicine Research Ethics Committee, University of Malawi. The CASTLE trial is registered with clinicaltrials.gov (NCT04545164). CASTLE was funded by Wellcome (203905/Z/16/Z). The funder had no role in the study design, data collection, data analysis, data interpretation, or writing of manuscript. All participants gave written or witnessed thumbprint informed consent. Anonymized trial data and analysis code are available at London School of Hygiene & Tropical Medicine Data Compass.

## RESULTS

**Figure 1.**
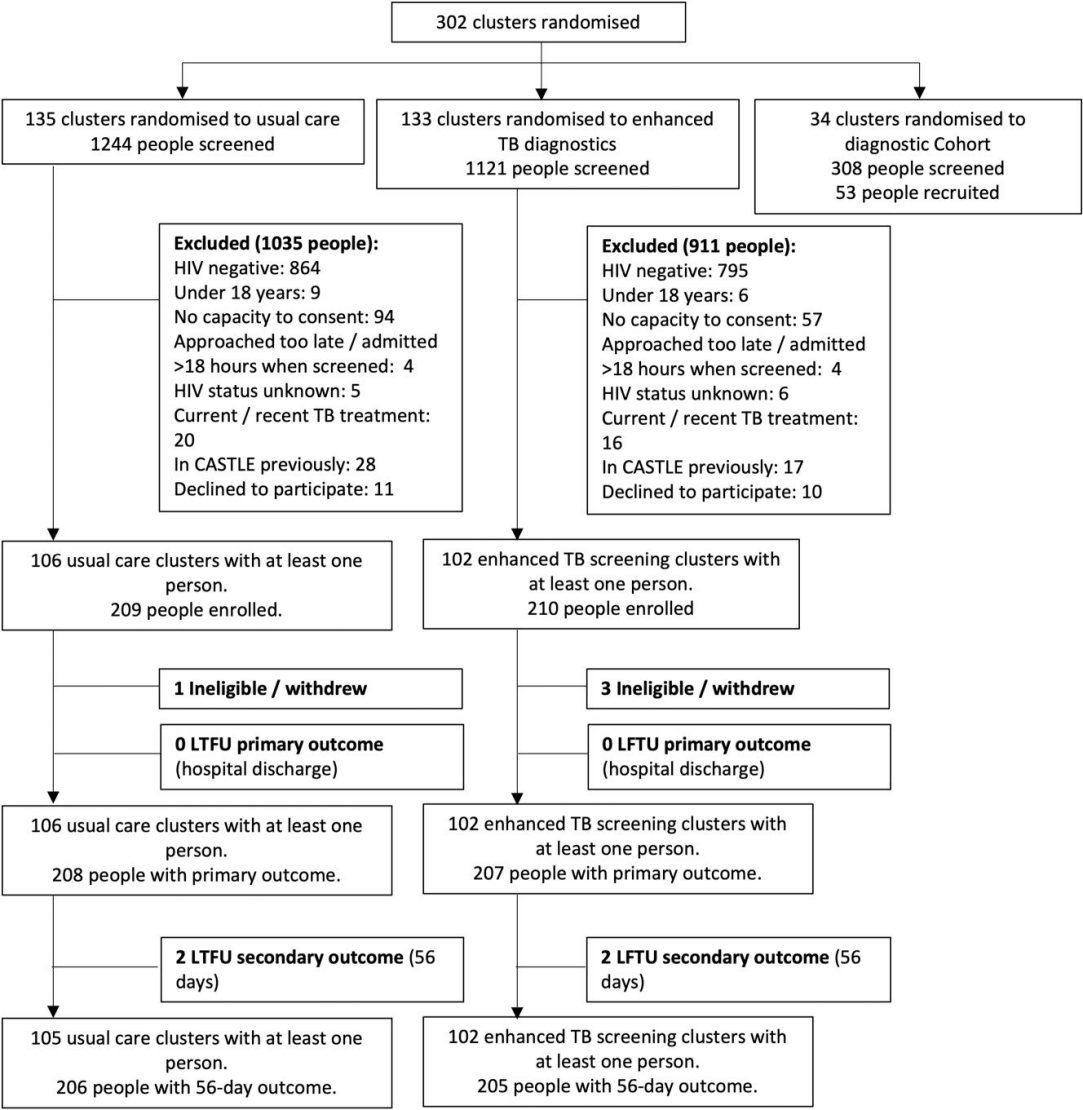
CONSORT diagram.

Between 2 September 2020 and 15 February 2022, we screened 2673 people admitted to adult medical wards and recruited 419 trial participants in 208 clusters (admission days, see Supplementary Table 1 for further details). A further 53 participants (28 clusters with at least 1 person) were recruited on days randomly assigned to the diagnostic cohort and will be described separately. Three participants were excluded after recruitment because of ineligibility and 1 participant withdrew, leaving 415 participants in the modified intention-to-treat analysis (Figure 1).

Baseline characteristics are shown in Table 1. The median age was 41 years (interquartile range: 36–51 years) and 56.6% (235/415) were female. Most participants (78.6%, 326/415) had been taking ART for more than 6 months, and 18.9% (58/307) of people who had a CD4 cell count measured had values below 100 cells/mm^3^.

**Table 1.** Characteristics of Clusters and Participants by Trial Arm

	Usual Care Arm	Enhanced TB Diagnostics Arm
Clusters		
Number of clusters randomized (including clusters in which no one was admitted)	135	133
Number of clusters included (clusters where at least 1 person was recruited)	106	102
Median size of cluster (min.–max.)	2 (1–5)	2 (1–5)
Participants		
Number of participants	208	207
Age in years (median, IQR)	42 (36–51)	41 (32–51)
Sex		
Men	84 (40.4%)	96 (46.4%)
Women	124 (59.6%)	111 (53.6%)
ART status		
Not on ART (new HIV diagnosis)	19 (9.1%)	14 (6.8%)
Interrupted ART	5 (2.4%)	2 (1.0%)
On ART <6 mo	20 (9.6%)	29 (14.0%)
On ART ≥6 mo	164 (78.8%)	162 (78.3%)
TB symptoms (self-reported)		
Cough	60 (28.8%)	62 (30.0%)
Fever	64 (30.8%)	65 (31.4%)
Night sweats	45 (21.6%)	41 (19.8%)
Weight loss	96 (46.2%)	82 (39.6%)
Any ≥1 TB symptom	148 (71.2%)	139 (67.1%)
TB in differential diagnosis at admission^a^	62 (29.8%)	70 (33.8%)
Cannot walk unaided	105 (50.5%)	105 (50.7%)
CD4 cell count^b^		
Median cell/mm^3^ (IQR)	288 (146–438)	220 (110–440)
<100 cells/mm^3^	27 (13.0%)	31 (15.0%)
≥100 cells/mm^3^	131 (63.0%)	118 (57.0%)
Not measured/not recorded	50 (24.0%)	58 (28.0%)

Abbreviations: ART, antiretroviral therapy; HIV, human immunodeficiency virus; IQR, interquartile range; TB, tuberculosis.

^a^Tuberculosis in differential diagnosis at admission means the admitting (nonstudy) clinician had written tuberculosis as a possible diagnosis in the medical record (ie, the admitting clinician suspected TB). This was determined from admission medical notes review before and diagnostic tests were done).

^b^CD4 counts provided by the routine health service.

The median duration of hospital stay was 6 days (interquartile range: 3–9 days). By hospital discharge, 16.9% (70/415) participants had started TB treatment: 11.5% (24/208) in the usual care arm and 22.2% (46/207) in the enhanced TB diagnostics arm. The risk ratio for starting TB treatment was 1.92 (95% confidence, 1.20–3.08) for intervention versus usual care participants. The intra-cluster correlation coefficient was 0.088. Results were similar in prespecified subgroups analyses defined by CD4 count and whether TB was in the differential diagnosis at admission (Table 2).

**Table 2.** Effect of Intervention on Trial Outcomes

	Usual Care Arm	Enhanced TB Diagnostics Arm	Relative Risk (95% CI)	Absolute Risk Difference (Percentage Points, 95% CI)
Primary outcome				
TB treatment initiation during admission	24/208 (11.5%)	46/207 (22.2%)	1.92 (1.20–3.08)	+10.7 (+3.34 to +18.0)
TB treatment initiation by subgroups				
TB was in differential diagnosis at admission	12/62 (19.4%)	19/70 (27.1%)	1.40 (.76–2.60)	+7.79 (−5.88 to +21.4)
TB not in differential diagnosis at admission	12/146 (8.2%)	27/137 (19.7%)	2.34 (1.24–4.64)	+11.5 (+2.95 to +20.0)
CD4 count categories				
<100 cells/mm^3^	5/27 (18.5%)	10/31 (32.3%)	1.74 (.67–4.52)	+13.7 (−8.76 to +36.1)
≥100 cells/mm^3^	16/131 (12.2%)	27/118 (22.9%)	1.87 (1.05–3.34)	+10.7 (+1.30 to +20.0)
CD4 not measured	3/50 (6.0%)	9/58 (15.5%)	2.59 (.74–8.93)	+9.52 (−1.71 to +20.7)
Secondary outcomes				
Undiagnosed TB at discharge^a^	2/208 (1.0%)	0/207 (0.0%)	Not estimated^a^	Not estimated^a^
TB treatment within 24 h of admission	5/208 (2.4%)	8/207 (3.9%)	1.61 (.54–4.81)	+1.46 (−1.86 to +4.79)
Death by 56 d	52/208 (25.0%)	54/207 (26.1%)	HR 1.05 (.72–1.53)^b^	NA^b^

Abbreviations: CI, confidence interval; HR, hazard ratio (time to event outcome for death); NA, not applicable; TB, tuberculosis.

^a^Not estimated because there were no events in the intervention arm. *P* value (Fisher exact) for difference between arms is .50.

^b^Not applicable because absolute risk not meaningful for a time-to-event outcome.

**Figure 2.**
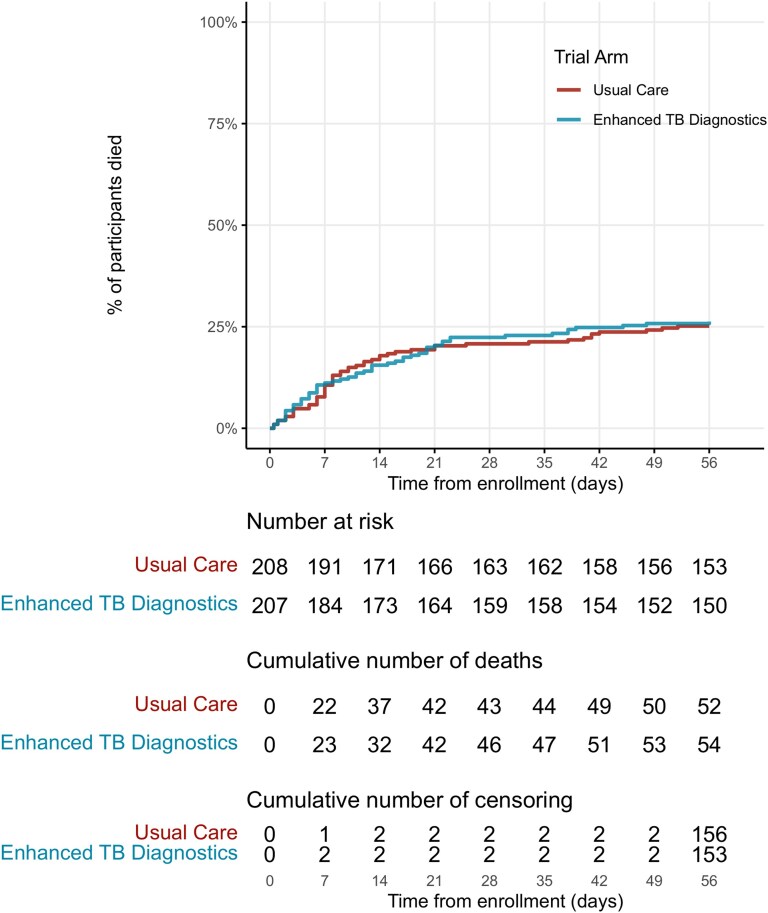
Survival curve showing hazard of death by trial arm.

By 56 days from enrollment, 4 participants were lost to follow-up: 2 in the usual care arm and 2 in the enhanced TB diagnostics arm. There was no difference in mortality, undiagnosed TB at discharge, or TB treatment with 24 hours of enrollment between the 2 trial arms (Table 2 and Figure 2).

We obtained dCXR-CAD scores for 96.6% (200/207) and SILVAMP-LAM results for 97.1% (201/207) of participants in the enhanced TB diagnostics arm. CAD4TB scores were generally high: 51% (102/200) had a CAD4TB score ≥60 (see Supplementary Appendix Figure 1).

At least 1 Determine-LAM was performed in 38.5% (80/208) of participants in the usual care arm and in 97.1% (201/207) participants in the enhanced TB diagnostics arm. Sputum Xpert tests were done on 20.2% (42/208) people in the usual care arm and 40.6% (84/208) in the enhanced TB diagnostics arm, including 66% (67/102) participants with CAD4TB score ≥60. Seven sputum Xpert tests were positive. Sputum for cultures was taken on admission from 53.4% (111/208) and 55.6% (115/207) people in the usual care and enhanced TB diagnostics arms, respectively. Eight participants had a positive sputum culture on study admission sample, 4 in each arm (8/415, 1.9% of all participants or 8/226, 3.5% of all participants with sputum sent for culture).

**Figure 3.**
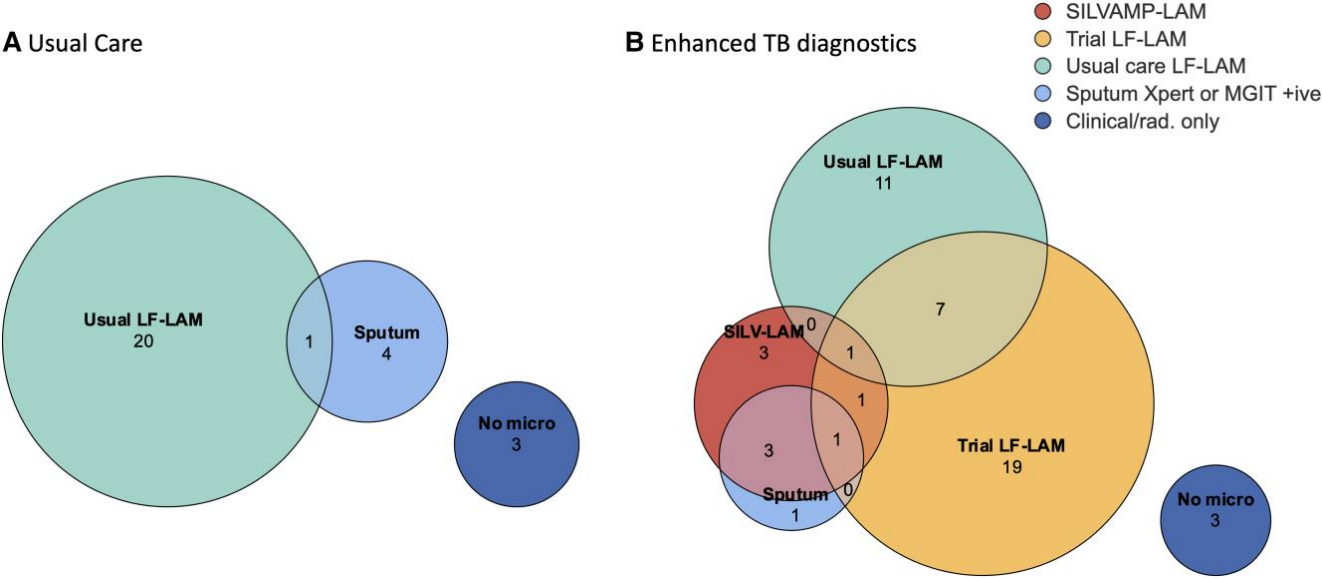
Positive TB tests. (*A*) Usual care arm and (*B*) enhanced TB diagnostics arm. Determine-LAM, Determine Determine-LAM urine test manufactured by Alere/Abbott (USA); SILVAMP-LAM, SILVAMP LAM urine test manufactured by FujiFilm (Japan); Xpert, Xpert Mtb/rif rapid molecular diagnostic test, manufactured by Cephid (USA); Clinical/rad means clinical or radiological diagnosis only (no microbiological diagnosis). Only, participants who started TB treatment based on clinical or radiological findings, but without positive microbiological tests. Trial Determine-LAM means Determine-LAM testing done by CASTLE trial team. Usual Determine-LAM means Determine-LAM done by usual care team. Results shown for all participants who had at least 1 positive TB test or who started TB treatment. Includes 8 people (4 in each arm) who had a positive test but did not start TB treatment.

We observed substantial discrepancies between results of Determine-LAM and SILVAMP-LAM testing. Overall, 14.4% (29/201) Determine-LAM tests done by the study team (ie, not including “usual care” Determine-LAM tests) were positive and 4.5% (9/201) SILVAMP-LAM tests were positive, with poor concordance between tests (Table 3). Of the 6 people who had a positive urine test by SILVAMP-LAM but negative by Determine-LAM, 3 had TB confirmed by a sputum microbiological test. Figure 3 summarizes microbiological basis for TB diagnosis in each trial arm, including for 8 people (4 in each arm) who had positive TB tests but did not start TB treatment. Further details are in Supplementary Appendix Tables 2, 3, 4, 5, and 6 and Supplementary Figure 2.

**Table 3.** Urine Lipoarabinomannan Results in the Enhanced TB Diagnostics arm Participants

		Urine SILVAMP LAM Results^a^				
		Positive	Negative	Indeterminate	Not done	Totals
Urine determine-LAM results	Positive grade 2–4	1	6	0	0	7
	Positive grade 1	2	20	0	0	22
	Negative	6	165	1	0	172
	Not done	0	0	0	6	6
Totals	…	9	191	1	6	207

Abbreviation: LAM, lipoarabinomannan.

^a^Results of testing fresh urine, both tests conducted on the same sample.

## DISCUSSION

In this cluster randomized trial, PHIV admitted to hospital on days randomized to enhanced TB diagnostics had significantly increased likelihood of starting TB treatment during admission (22.2%) compared to usual care (11.5%), but no reduction in the high risk of death by 56 days from enrollment (25.0% usual care and 26.1% enhanced TB diagnostic arms). Enhanced TB diagnostics consisted of systematic provision of 2 urine LAM antigen tests (SILVAMP-LAM and Determine-LAM) and a dCXR-CAD on admission, followed by sputum Xpert MTB/RIF for people with chest X-ray abnormalities suggestive of TB. In the usual care arm, all TB tests normally available at the hospital (including conventional chest X-ray, sputum Xpert, and Determine-LAM) could be requested by treating clinicians. Most of the increase in TB diagnosis appeared to be driven because of greater use of World Health Organization–recommended Determine-LAM in the enhanced diagnostics arm and underuse of Determine-LAM in the usual care arm. We found that SILVAMP-LAM testing yielded substantially fewer positive results than Determine-LAM.

Since the CASTLE trial finished, 2 large studies of SILVAMP-LAM diagnostic accuracy [22, 23] that were running concurrently with CASTLE have published findings showing unexpected lot-to-lot variability of SILVAMP-LAM. As in CASTLE, poor concordance between Determine-LAM and SILVAMP-LAM was seen [23]. The batch of SILVAMP-LAM mostly used in CASTLE (#20004) was found to have high specificity but low sensitivity in 1 study [22]. Batch #19002 was less specific and more sensitive than other batches in a post hoc laboratory analysis. As a result of these large studies and CASTLE, it seems unlikely that this present iteration of SILVAMP-LAM will be commercially developed further.

In CASTLE, only 37% of participants in our usual care arm had urine Determine-LAM tests performed, despite this being recommended for all PHIV admitted to hospital in Malawi [24]. Among the subset of people who had Determine-LAM by usual care and by CASTLE, there were frequent discrepant results—the reasons for which are unclear. Quality assurance for urine Determine-LAM testing has not been optimized. Unlike point-of-care HIV rapid tests, manufacturers do not supply control material that can be used for quality control testing, there are no well-established or widely used standards for proficiency testing. Interpreting results for Determine-LAM relies on visually comparing a color band on a test strip to a band on a reference card, which might introduce subjective interpretations. Our results and multicountry evaluations of SILVAMP-LAM showing discordance between LAM tests results, underscore the urgent need for routine proficiency and quality assurance systems for LAM-based tests (perhaps including smartphone based automated readers), if LAM-based tests are to be scaled up more widely and interpreted with confidence.

To our knowledge, CASTLE was the first trial to investigate systematic testing with digital CXR-CAD for hospitalized PHIV, for whom the pretest probability of pulmonary TB is high, but so is the probability of other pulmonary infections and radiological abnormality not due to TB disease [25]. The software, CAD4TB, should be considered a useful adjunct to be used alongside specific diagnostic tests and clinical judgment, and not a replacement for either.

TB has consistently been shown to be the leading cause of both hospital admission and death in African adults with HIV [5–7]. In autopsy studies, TB is often undiagnosed at the time of death. Although systematic Determine-LAM testing has been shown to reduce all-cause mortality, randomized trials in outpatient settings that have investigated the impact of sputum Xpert testing have mostly failed to show morbidity or mortality benefit [26]. A trial of enhanced TB diagnostics in hospitalized children with severe pneumonia showed no mortality benefit [27]. As such, there is still considerable uncertainty as to the optimal screening, diagnostic, and management strategies for providing early TB diagnosis for inpatients and outpatients with advanced HIV disease, suggesting the need for more intervention trials with patient-important outcomes beyond diagnostic accuracy. Urine LAM tests can detect people at the highest risk of mortality from TB—in addition to accurate diagnosis, improvements in treatment might be needed to further reduce mortality.

Comparing CASTLE trial participants with a previous trial of TB screening (STAMP: 2015–2017) recruiting at the same hospital and with the same eligibility criteria, we observed lower number of daily admissions to medical wards for PHIV, fewer people not on ART (20% STAMP, 8% CASTLE), but relatively similar low CD4 counts (median CD4 219 cells/mm^3^ STAMP, 240 cells/mm^3^ CASTLE). Although progress has been made between 2015 and 2020 with expanding ART treatment in Malawi, little has changed regarding high risk of death for people admitted to hospital (22% STAMP, 25% CASTLE) [1, 11]. To achieve goals of ending acquired immunodeficiency syndrome, more focus and evidence-based interventions on preventing deaths from advanced HIV are needed [1,2].

CASTLE was a pragmatic and relatively small single-site trial, designed to provide preliminary estimates of the likely impact of introducing 2 TB diagnostic interventions with high potential to be scaled up. Limitations include a sample size that was underpowered for our secondary outcome of death within 56 days. We observed far less than expected agreement between TB tests, with most patients started on treatment due to otherwise unconfirmed Determine-LAM positive results. The coronavirus disease 2019 pandemic likely affected both our case mix and the diagnostic performance of dCXR-CAD in ways that cannot be clearly quantified and no longer apply. We also note a relatively low prevalence of culture-confirmed TB diagnosed through sputum culture, with only 3.5% of specimens being culture positive. By comparison, 8.1% of sputum Xpert tests were positive when sputum was collected in a similar systematic way from STAMP trial participants. Xpert MTB/RIF was in use in Malawi during 2020–2022; if Ultra had been used instead, it is possible more TB would have been detected. We expect a high proportion of people with TB in this group to have extrapulmonary or disseminated TB, so that sputum Xpert does not reliably exclude TB. Population-wide TB incidence is declining in Malawi, and it is possible that the true burden of TB as a cause of admission among PHIV has indeed declined since 2015 and—if true—this might alter the risks and benefits associated with systematic TB screening.

Despite these limitations, we have shown that systematic testing with DCXR-CAD and 2 urine LAM tests is feasible. The lack of impact on mortality is disappointing, given that short-term outcomes for adults with HIV admitted to hospitals in Southern and Eastern Africa remain extremely poor [1–3, 28, 29]. Assuming that TB remains the major cause of hospital admission and death, quality-assured TB diagnostics need to be developed and made available for inpatients, to support high-quality clinical care including clinician judgment about empiric TB treatment. Existing tools—including Determine-LAM—need to be fully implemented and appropriately supported. More research is urgently needed to understand causes of death in the context of ART scale-up, define appropriate packages of interventions for people with advanced HIV in hospital, support evidence-based interventions, and provide clearer treatment guidelines focused on severe bacterial infections, TB, and ART treatment failure.

## Supplementary Material

ciae273_Supplementary_Data

## Supplementary Data

Supplementary materials are available at *Clinical Infectious Diseases* online. Consisting of data provided by the authors to benefit the reader, the posted materials are not copyedited and are the sole responsibility of the authors, so questions or comments should be addressed to the corresponding author.

## Note

***Financial support*.** CASTLE was funded by Wellcome (203905/Z/16/Z). The funder had no role in the study design, data collection, data analysis, data interpretation, or writing of the manuscript.
