# Supplementary material for: Enhanced Tuberculosis Diagnosis With Computer-aided Chest X-ray and Urine Lipoarabinomannan in Adults With HIV Admitted to Hospital (CASTLE Study): A Cluster Randomized Trial
Source: Clin Infect Dis. 2024 May 15;80(5):1143–51. doi: 10.1093/cid/ciae273 (PMC12135911; doi:10.1093/cid/ciae273)
Supplement: ciae273_Supplementary_Data [file ciae273_supplementary_data.pdf]

CASTLE trial appendix

Supplementary methods

Details on clusters and timing of recruitment

Each cluster was an admission day, covering a 24-hour period between 3:00pm and 2:59pm.

Screening and recruitment happened during office hours: people admitted to hospital in the late afternoon (after 3pm) or overnight could be recruited the following morning, but people admitted at weekends (before 3pm on Sunday) or before 3pm on public holidays were not recruited.

Each day's allocation was revealed by opening the envelope at 8am each morning. Participants admitted the previous evening (after 3pm) and during that day (up to 2:59pm) received the intervention according to the allocation.

Details on urine LAM testing

For participants in the enhanced TB diagnostics arm, urine LAM tests (SILVAMP-LAM and Determine-LAM) were performed by CASTLE study staff, in a study room adjacent to medical ward. Study staff were provided with training in conducting and interpreting LAM results (Determine-LAM and SILVAMP-LAM) with regular supervision. Urine LAM (both SILVAMP-LAM and Determine-LAM) results were read by two study staff members to ensure consistency of judgement, although they weren't blinded to each other's reading. These results were recorded on a paper trial ledger, and the electronic study database in real time. They were also reported into participants medical records for use by routine clinical staff.

Determine-LAM (but not SILVAMP-LAM) was also available through usual care. In this case, Determine-LAM was conducted the routine clinical staff (not CASTLE staff) using their standard operating procedures. CASTLE were not involved in training or supervising Determine-LAM tests done by routine staff team. The results for these Determine-LAM tests were recorded in paper ledger from the Malawi Department of HIV/AIDS (not the CASTLE ledger). Results were collected from this ledger retrospectively periodically during CASTLE trial. For people in usual care arm, their only access to Determine-LAM was through usual care. For people in enhanced TB diagnostics arm some participants had an Determine-LAM result by both the CASTLE team, and by routine care team.

Table S1: Reasons for non-randomisation of clusters (days).

Between 2nd September 2020 and 15th February 2022 there were 532 days (inclusive). In total, 302 days were randomised (see CONSORT diagram), although not all of these became clusters because in some days no eligible participants were recruited.

In total, 1312 people were admitted to hospital at weekends, public holidays or other days that were not eligible for randomisation to a study cluster.

Reasons for non-randomisation of days and number of admissions on those days were as follows:

	Number of days not randomised	Number of people admitted on those days
Christmas period 2020 and 2021	28	Not measured
COVID study close (Jan 2021)	16	190
Saturday or Sunday	144	839
Friday before May 2021*	32	240
Public holidays (not including public holidays around Christmas and in Jan 2021)	10	43

* At the start of the CASTLE trial we didn't recruit on Fridays, to enable study team members to have time for administration and other tasks. Due to lower than anticipated recruitment, in May 2021, we amended the protocol to recruit on Fridays.

Table S2: TB tests performed

	**Usual care arm**	**Enhanced diagnostics arm**
dCXR with CAD score [(a)]	0 (0.0%)	200 (96.6%)
Urine SILVAMP-LAM	0 (0.0%)	201 (97.1%)
Urine Determine – LAM [(b)]	80 (38.5%)	201 (97.1%)
Sputum Xpert	42 (20.2%)	84 (40.6%)
Sputum culture	111 (53.4%)	115 (55.6%)

Number of participants with at least one of the following TB test performed. dCXR-CAD = Digital Chest X-ray with Computer Aided Diagnosis, SILVAMP-LAM = SILVAMP LAM urine test manufactured by FujiFilm (Japan), Determine-LAM = Determine Determine-LAM urine test manufactured by Alere/Abbott (USA). Xpert = Xpert Mtb/rif rapid molecular diagnostic test, manufactured by Cephid (USA).
[(a)] It was not possible to determine how many people had a conventional (non-digital) chest x-ray.
[(b)] Includes either Determine-LAM performed by trial staff or usual care.

Table S3: Urine LAM results by SILVAMP-LAM batch number

		**Determine-LAM**		**Overall SILVAMP** (by batch)
		Positive (any grade)	Negative	
**SILVAMP LAM**				
Batch 19002 (n=51 tests)	Positive	2	2	**4/51 (8%)**
	Negative	9	38	47/51(92%)
Batch 20004 (n=151 tests)	Positive	1	4	**5/150 (3%)**
	Negative	17	127	144/150 (96%)
	Indeterminate	0	1	1/150 (1%)
	**Overall Determine-LAM**	29/201 (14%)	172/201 (86%)	

Table S4: Sputum Culture Results

**Usual care arm**				
	**Sputum Xpert**			
**Sputum Culture (MGIT)**	a) Xpert negative	b) Xpert positive	c) Xpert not done	**TOTAL (MGIT culture)**
a) Negative	25	1	72	98
b) *M. tb*	1	1	2	4
c) MOTT	0	0	1	1
d) Contaminated	4	0	4	8
e) No sputum received in lab for culture.	10	0	87	97
**Enhanced TB diagnostics arm**				
	**Sputum Xpert**			
**Sputum Culture (MGIT)**	a) Xpert negative	b) Xpert positive	c) Xpert not done	**TOTAL (MGIT culture)**
a) Negative	54	0	38	92
b) *M. tb*	0	4	0	4
c) MOTT	0	0	0	0
d) Contaminated	10	1	8	19
e) No sputum received in lab for culture.	15	0	77	92

MOTT = Mycobacterium other than tuberculosis (not possible to provide further species information)

Table S5: Usual care diagnostic use by CD4 count (A,C) and TB in differential diagnosis at admission (B,D)

**A: CD4**	**Determine LAM done by usual care?**				
	**Determine LAM not tested**	**Determine LAM tested**			**Total**
**CD4 count category**		**LAM tested (overall)**	**Positive**	**Negative**	
a) Under 100 cells/mm^3	19 (33%)	39 (67%)	10 (17%)	29 (50%)	58 (100%)
b) 100 to 200 cells/mm^3	21 (32%)	45 (68%)	10 (15%)	35 (53%)	66 (100%)
c) 200 or more cells/mm^3	115 (63%)	68 (37%)	15 (8%)	53 (29%)	183 (100%)
d) No CD4 measured	99 (92%)	9 (9%)	5 (5%)	4 (4%)	108 (100%)
Total	254	161	40	121	415

**B: TB Suspect**	**Determine LAM done by usual care?**				
	**Determine LAM not tested**	**Determine LAM tested**			**Total**
**TB in differential diagnosis at admission?**		**LAM tested (overall)**	**Positive**	**Negative**	
a) Yes	62 (47%)	70 (53%)	16 (12%)	54 (41%)	132 (100%)
b) No	192 (68%)	91 (32%)	24 (8%)	67 (24%)	283 (100%)
Total	254	161	40	121	415

**C: CD4**	**Sputum Xpert done at hospital lab?***				
	**Sputum Xpert not tested**	**Sputum Xpert tested**			**Total**
**CD4 count category**		**Xpert tested (overall)**	**Xpert positive**	**Xpert negative**	
a) Under 100 cells/mm^3	31 (53%)	27 (46%)	2 (3%)	25 (43%)	58 (100%)
b) 100 to 200 cells/mm^3	40 (61%)	26 (40%)	1 (2%)	25 (38%)	66 (100%)
c) 200 or more cells/mm^3	131 (72%)	52 (29%)	3 (2%)	49 (27%)	183 (100%)
d) No CD4 measured	87 (81%)	21 (20%)	1 (1%)	20 (19%)	108 (100%)
Total	289	126	7	119	415

**D: TB suspect**	**Sputum Xpert done at hospital lab?***				
	**Sputum Xpert not tested**	**Sputum Xpert tested**			**Total**
**TB in differential diagnosis at admission?**		**Xpert tested (overall)**	**Xpert positive**	**Xpert negative**	
a) Yes	67 (51%)	65 (49%)	5 (4%)	60 (45%)	132 (100%)
b) No	222 (78%)	61 (22%)	2 (1%)	59 (21%)	283 (100%)
Total	289	126	7	119	415

* Includes sputum Xpert requested by CASTLE team and by usual care, all were processed in same lab and results released on same computer system / ledger.

Table S6: Death by LAM and TB treatment status

	Outcome at 56 days			
	a) Died	b) Alive	c) Lost to follow up	Total
TB / LAM category				
a) TB treated, LAM neg*	4 (29%)	10 (71%)	0 (0%)	14 (100%)
b) TB treated, LAM pos**	21 (38%)	35 (62%)	0 (0%)	56 (100%)
c) No TB diagnosed	77 (23%)	257 (76%)	3 (1%)	337 (100%)
c) Other***	4 (50%)	3 (38%)	1 (12%)	8 (100%)

* includes 4 people (1 died, 3 alive) who had TB diagnosed and LAM not done
** Includes people with positive urine LAM test on one or more of Determine LAM performed by CASTLE staff, Determine LAM performed by usual care staff or SILVAMP-LAM (all SILVAMP LAM by CASTLE staff)
*** These 8 people were people who had a TB test positive, but were not diagnosed with TB – either because they died before TB treatment could be started or because the clinicians chose not to treat despite the positive test result.

Figure S1: CAD4TB scores

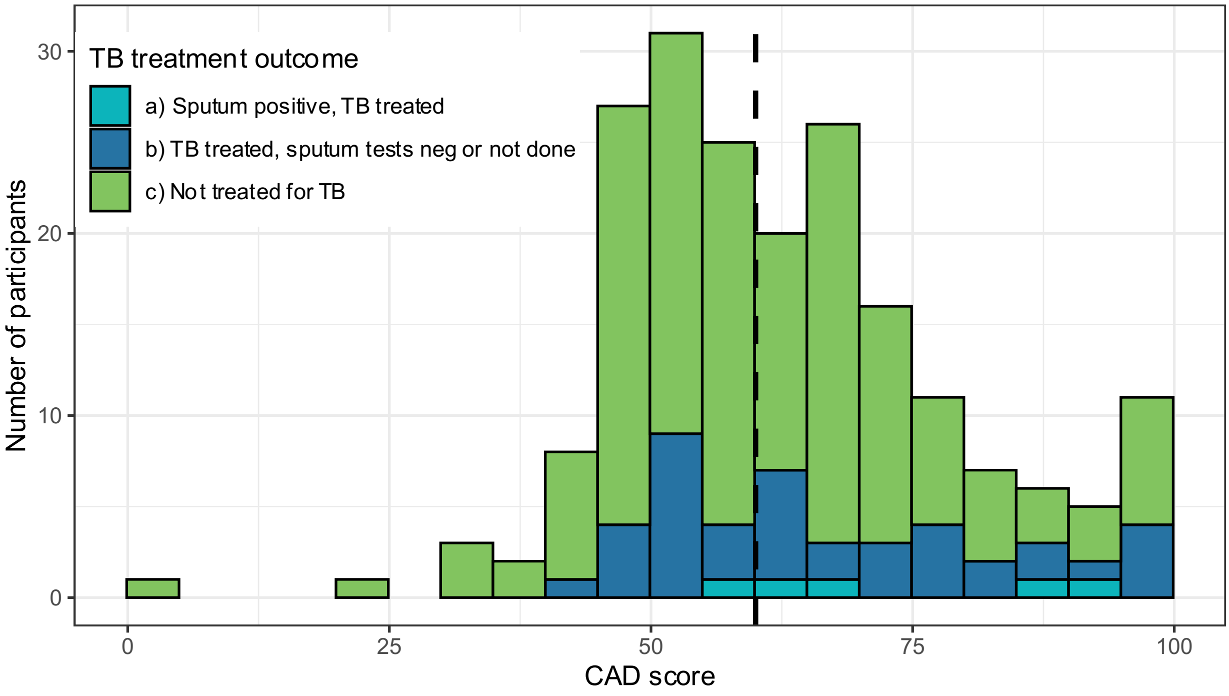

CAD4TBv6 scores for 200 people in Enhanced TB diagnostics arm who had a valid score.

Figure S2: TB tests performed and results

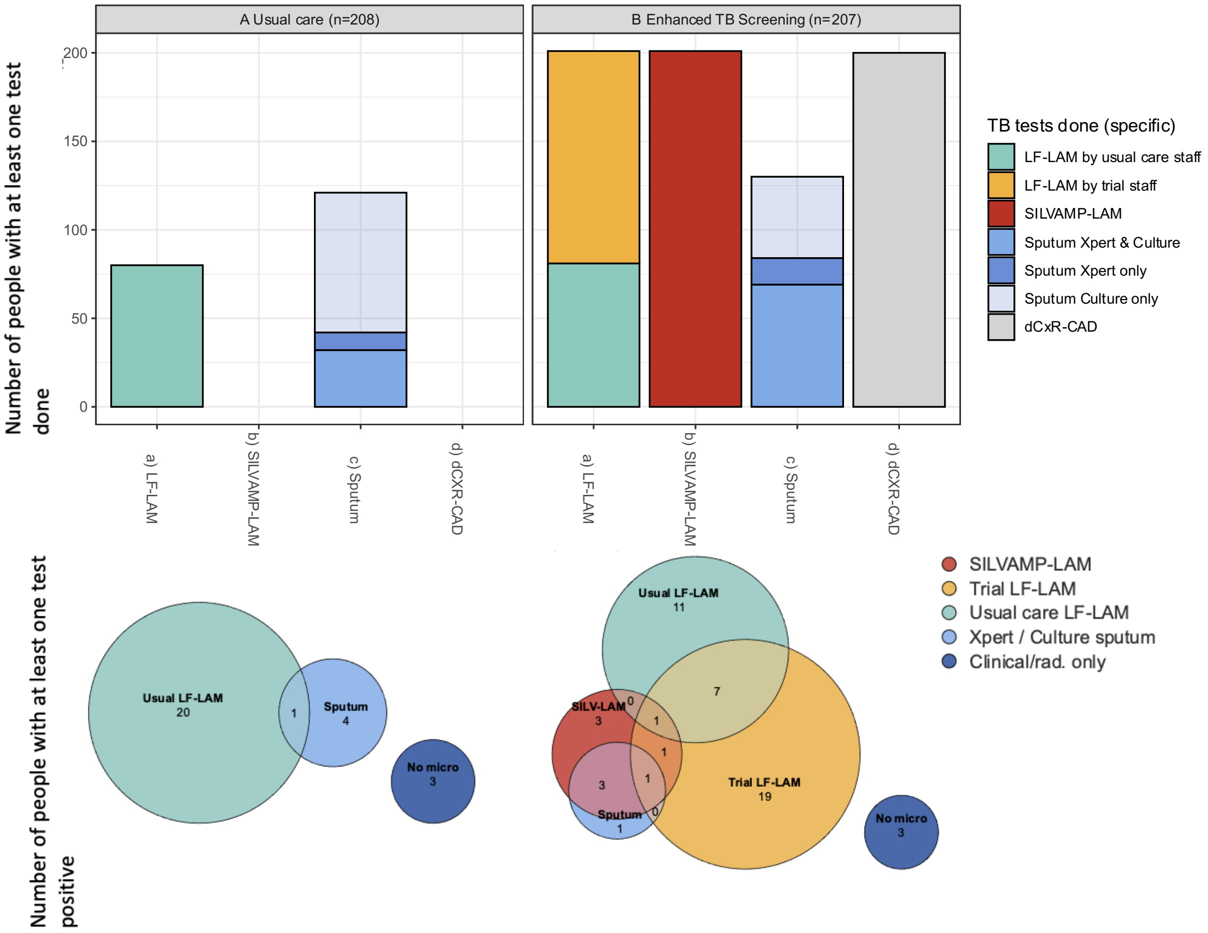

"Sputum" circle contains Xpert and Culture results, combined together for ease of understanding Euler diagram.

Five people in each arm had at least one positive sputum test. Note that where multiple sputum tests were done – culture and Xpert – these were done on different sputum samples. In the usual care arm the five people with positive sputum results were as follows: one culture positive + Xpert positive, two culture positive + Xpert not done, one culture positive + Xpert negative and one culture negative + Xpert positive. In the enhanced TB diagnostics arm the five positive sputum results were as follows: four people had culture positive + Xpert positive and one person culture contaminated + Xpert positive.
